# An Analysis Regarding the Prognostic Significance of MAVS and Its Underlying Biological Mechanism in Ovarian Cancer

**DOI:** 10.3389/fcell.2021.728061

**Published:** 2021-10-14

**Authors:** Lifeng Chen, Jing Hou, Bingbing You, Feifei Song, Xinyi Tu, Xiaodong Cheng

**Affiliations:** ^1^Department of Gynecologic Oncology, Women’s Hospital, School of Medicine, Zhejiang University, Hangzhou, China; ^2^Department of Gynecology, Zhejiang Provincial People’s Hospital, Hangzhou, China; ^3^Department of Breast Surgery, Guizhou Provincial People’s Hospital, Guiyang, China; ^4^Department of Molecular Pharmacology and Experimental Therapeutics, Mayo Clinic, Rochester, MN, United States

**Keywords:** MAVS, immunohistochemistry, immune cells, sensitivity analysis, ovarian cancer

## Abstract

The present study evaluates the value of mitochondrial antiviral signaling (MAVS) expression as a potential diagnostic biomarker and therapeutic target for ovarian cancer (OC) and analyses the underlying biological mechanism in this pathology. First, the association between MAVS expression determined by immunohistochemical (IHC) and clinical characteristics was systematically investigated. Overexpression of MAVS was associated with advanced clinical factors and poor survival of OC patients. Second, bioinformatics analyses, namely, gene expression, mutation analysis, gene set variation analysis (GSVA), gene set enrichment analysis (GSEA), and weighted gene co-expression network analysis (WGCNA), were performed to evaluate the potential biological functions of MAVS in OC. The results showed that MAVS may play a critical role in immune cell infiltration. CIBERSORT was applied to assess the infiltration of immune cells in OC. CD8+ T cells, γδT cells, and eosinophils had significantly negative correlations with MAVS expression. Finally, sensitivity analysis found that patients with high MAVS expression were predicted to be significantly less responsive to cisplatin and paclitaxel. In conclusion, these findings suggested that MAVS influences biological behavior by regulating the immune response and that it can be used as a predictive marker for poor prognosis in OC.

## Introduction

Ovarian cancer (OC) represents the leading cause of death from gynecologic malignancies. It is estimated that 21,410 new cases of OC are expected in 2021 with more than 13,770 deaths in the United States (Siegel et al., 2021). Recent progress in understanding the role of the immune system in cancers has simultaneously promoted the development of immunotherapies for specific cancer types. However, limited analysis of immunotherapy for ovarian cancer has been reported in recent years. Therefore, identification of a novel immune indicator to predict prognosis or development of targeted therapy for OC patients will contribute to the application of immunotherapy for OC.

Mitochondrial antiviral signaling (MAVS) protein, also known as virus-induced-signaling adapter (VISA) or IFN-β promoter stimulator protein 1 (IPS-1), is located on the mitochondrial membrane and is critical to innate immune defense against viral infection. It has also been suggested that MAVS is highly related to apoptosis in cancer cells (Matsushima-Miyagi et al., 2012; Kaneda, 2013; Pythoud et al., 2015). In colon cancer, MAVS activation exhibits tumor-suppressive activity by regulating p53 protein stability (Zhang W. et al., 2020). However, there is no evidence showing the correlation between MAVS and OC based on clinical data. Therefore, the detailed biological functions of MAVS need systematic investigation.

In the present study, the correlation between MAVS expression and clinical characteristics was analyzed based on 128 OC tissues. The underlying functions and mechanisms of MAVS in OC were then further investigated by performing gene expression analysis, mutation analysis, gene set variation analysis (GSVA), gene set enrichment analysis (GSEA), weighted gene co-expression network analysis (WGCNA), immune infiltration, and chemosensitivity prediction based on the OC dataset downloaded from The Cancer Genome Atlas (TCGA).

## Materials and Methods

### The Cancer Genome Atlas Data Download

mRNA and miRNA expression profile and clinical sample data of ovarian cancer were downloaded from TCGA official website^1^. Totally, the data of 379 ovarian cancer patients were analyzed.

### Tissue Microarrays and Immunohistochemistry

Tissue microarrays contained 128 ovarian cancers with clinical characteristics were purchased from Alenabio and Shanghai Outdo Biotech Company. Immunohistochemical (IHC) staining was carried out as previously described (Chen et al., 2021). All samples were scored by three independent pathologists. Staining intensity and staining area percentage were used as the criteria for evaluation. Staining intensity was scored as: no staining, 0; slight staining, 1; moderate staining, 2; and strong staining, 3. Staining area percentage was scored as: negative, 0; <10% positive cells, 1; 11–50% positive cells, 2; 51–80% positive cells, 3; and >80% positive cells, 4. By multiplying the two scores above, we got the final score. Staining for MAVS was considered as negative when the score is less or equal to 2; otherwise, the expression was noted positive.

### Co-expression Analysis and Nomogram Prediction Analysis

The limma package in R program was performed to screen genes that were co-expressed with MAVS. The thresholds for co-expression were a correlation coefficient >0.4 and *P* < 0.001. Correlation plots of top genes positively and negatively associated with MAVS were carried by the heat map and corrplot package in R. Besides, nomogram prediction analysis with three features achieved the favorable prediction efficacy for OC following R analysis.

### Mutation Analysis

We downloaded the SNP-related data of ovarian cancer, and the mutation genes were obtained from the SNP data of VarScan. The genes with top 30 mutation frequency were selected as the display. The difference of mutation genes between the two groups with positive or negative MAVS expression was compared, and the landscape map of mutation was drawn with R package complex heat map.

### Gene Set Variation Analysis and Gene Set Enrichment Analysis

Gene set variation analysis is a non-parametric unsupervised method for evaluating the enrichment of transcriptome gene sets, which was used to find the pathways most associated with MAVS. Based on the expression of MAVS, samples were divided into high and low expression groups. The gene set was downloaded from the Molecular Signature Database. GSEA 4.0 software was used to explore the signal pathway differences between the positively and negatively MAVS expression groups, where |NES| > 1 and FDR < 0.05 were defined statistically significant, to explore the possible molecular mechanism of prognosis differences between the two groups.

### Weighted Gene Co-expression Network Analysis

The WGCNA R package was used to construct a weighted gene co-expression network and the top 5,000 genes were screened for further study. The weighted adjacent matrix was transformed into a topological overlap matrix (TOM) to estimate the network connectivity and hierarchical clustering was used to construct the clustering tree structure of the TOM matrix. Different gene modules were represented as different color branches of the cluster. Based on the weighted correlation coefficient of genes, genes with similar expression patterns are classified into one module.

### Analysis of Immune Infiltration

Ovarian cancer samples with positive and negative expression of MAVS were divided into two groups. The CIBERSORT was used to analyze RNA-seq data, and the relative proportion of 22 kinds of immune infiltrating cells was obtained. Spearman analysis was performed on MAVS expression and immune cell content. *P* < 0.05 was considered statistically significant.

### Prediction of Chemosensitivity

Genomics of Drug Sensitivity in Cancer (GDSC) is the largest publicly available pharmacogenomics database which can be used to predict response to anti-cancer drugs^2^. Base on this database, R package “pRRophetic” was carried to predict the chemosensitivity of each tumor sample. The regression method was used to estimate the IC50 of each specific chemotherapy drug. The prediction accuracy of IC50 was verified by 10-fold cross-validation with the GDSC training set. All parameter settings are default values.

### Statistical Analysis

Continuous variables were described as the mean ± SD. Categorical variables were reported using proportions. To compare the differences between the experimental groups, bilateral *t*-test, W2 test, or Kruskal–Wallis test as appropriate was adopted. Univariate analysis was used to assess the prognostic significance of the different variables. Variables with *P* value < 0.05 were further analyzed by Cox multivariate regression models. The relationship between overall survival and MAVS expression was presented with Kaplan–Meier survival curve and compared with a log-rank test. Data analysis and statistical evaluations were carried out by SPSS22.0 and GraphPad Prism 6. *P* value < 0.05 was considered statistically significant.

## Results

### Mitochondrial Antiviral Signaling Expression and Its Correlation With Clinical Parameters According to Immunohistochemical Analysis in Ovarian Cancer

**FIGURE 1:**
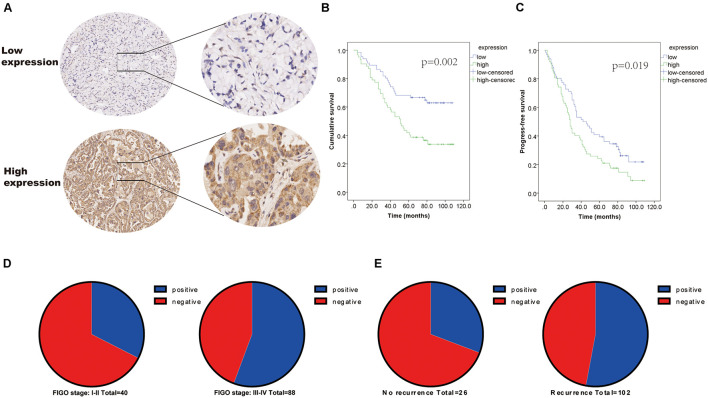
Positivity expression of MAVS is associated with advanced clinical factors and poor survival of OC patients. **(A)** Representative images of IHC staining of MAVS on tissue microarray of ovarian cancer specimens. **(B)** Overall survival (OS) of OC patients was analyzed by the Kaplan–Meier plotter analysis. **(C)** Progression-free survival (PFS) of OC patients was analyzed by the Kaplan–Meier plotter analysis. **(D)** MAVS expression in different clinical stages of OC was detected by IHC analysis. **(E)** MAVS expression OC patients with different status of recurrence was detected by IHC analysis.

The expression of MAVS in 128 ovarian cancer tissues was examined by immunohistochemistry (IHC). IHC staining indicated that MAVS expression was positive in 62 cases, accounting for 48.4% of ovarian cancers. The representative images of IHC staining of MAVS on tissue microarray of ovarian cancer specimens (Figure 1A). Table 1 presents the correlation between MAVS expression and clinicopathological characteristics. Positive MAVS expression was correlated with advanced International Federation of Gynecology and Obstetrics (FIGO) stage (55.7 vs. 32.5%, *P* = 0.015; Figure 1D), high Ki67 expression (61.2 vs. 34.0%, *P* = 0.003), high recurrence rate (55.2 vs. 29.6%, *P* = 0.017; Figure 1E), and PD-L expression (57.0 vs. 35.4%, *P* = 0.019). Univariate analysis found that the factors associated with both overall survival and progression-free survival were MAVS protein expression, FIGO stage, pathological grade, and tumor size (Table 2). Positive MAVS staining was correlated with lower OS and PFS compared to negative MAVS staining (*P* = 0.002; *P* = 0.016; Figures 1B,C).

**TABLE 1:** Correlation between MAVS expression and clinicopathological characteristics of OC patients.

	Variables	MAVS expression		Total	χ ^2^	*p* value
		Negative	Positive			
Age (year)					0.975	0.323
	≤50	34	27	61		
	>51	31	35	66		
FIGO stage					5.917	0.015
	I/II	27	13	40		
	III/IV	39	49	88		
Metastasis					1.824	0.176
	No	8	13	21		
	Yes	58	49	107		
Recurrence					4.078	0.043
	No	18	8	26		
	Yes	48	54	102		
Pathological grade					0.071	0.79
	I/II	16	18	34		
	III	31	39	70		
PD-L1 expression					5.547	0.019
	Negative	31	17	48		
	Positive	34	45	79		
Ki67 expression					8.78	0.003
	Negative	35	18	53		
	Positive	26	41	67		
Maximum size					1.208	0.272
	<12 cm	33	25	58		
	>12 cm	33	37	70		

*Statistically significant (*P* < 0.05).*

**TABLE 2:** Univariate and multivariate analysis of the factors correlated with **(A)** overall survival

Variables	Univariate analysis			Multivariate analysis		
	HR	95% CI	*p* value	HR	95% CI	*p* value
Expression	2.220	1.335–3.691	0.002	1.156	0.592–2.259	0.671
Grade	2.166	1.383–3.391	0.001	1.154	0.382–3.484	0.8
Age	1.407	0.852–2.325	0.182			
FIGO stage	8.539	3.194–22.829	0.000	5.768	1.343–24.78	0.018
Maximum size	4.92	2.683–9.024	0.000	3.097	1.453–6.601	0.003
Ki67	1.941	1.125–3.349	0.017	0.866	0.419–1.79	0.698
**(B) Progression-free survival of OC patients.**						
Expression	1.588	1.074–2.35	0.021	0.981	0.614–1.568	0.936
Grade	1.458	1.085–1.958	0.012	0.98	0.549–1.749	0.945
Age	1.71	1.147–2.548	0.008	1.163	0.700–1.932	0.561
FIGO stage	4.199	2.570–6.862	0.000	2.706	1.390–5.265	0.003
Maximum size	2.234	1.472–3.39	0.000	1.954	1.151–3.318	0.013
Ki67	1.386	0.921–2.086	0.118			

*Statistically significant (*P* < 0.05).*

### Data Acquisition

We downloaded the original mRNA expression data of ovarian cancer from the TCGA database. All samples were divided into high or low expression groups based on the expression of the MAVS gene with the median as a cutoff point.

### Co-expression Analysis and Prognostic Nomogram Construction of Mitochondrial Antiviral Signaling in Ovarian Cancer According to the Cancer Genome Atlas Data

**FIGURE 2:**
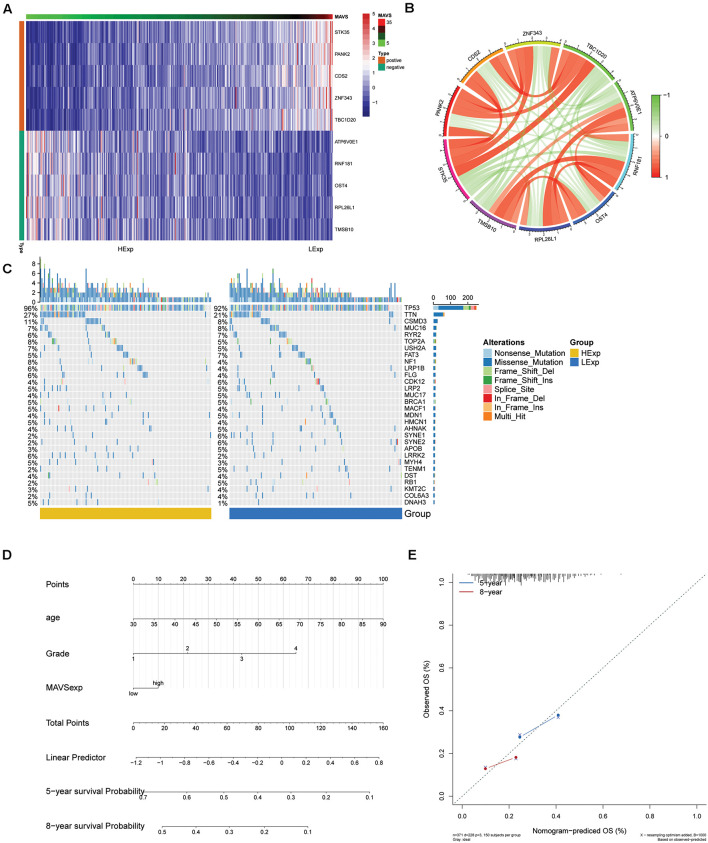
Significant MAVS-related genes or mutation genes in OC obtained by co-expression analysis and mutation analysis. **(A)** Heat map shows the top 10 genes that are differentially expressed in groups with positive or negative MAVS expressions of OC patients. **(B)** Network visualization of significant MAVS-related genes in groups with positive or negative MAVS expressions of OC patients. **(C)** Heat map of the MAVS-related mutation genes in OC obtained by mutation analysis. **(D)** Nomogram for predicting the 3- and 5-year survival rates of OC patients. **(E)** Calibration curve based on the 3-year OS of the nomogram.

According to the co-expression analysis, the top 15 genes positively and negatively associated with MAVS were identified using a heat map. In addition, the top five genes positively and negatively associated with MAVS were plotted in a circular plot. The results showed that MAVS was positively related to STK35, PANK2, CDS2, ZNF343, and TBC1D20 but negatively related to ATP6V0E1, RNF181, OST4, RPL26L1, and TMSB10 (Figures 2A,B). Furthermore, we conducted a nomogram prediction model according to the clinical symptoms and MAVS expression of patients. Five-year and 8-year survival rates for OC were predicted based on the nomogram score. The results showed that the model better predicted the survival rate of patients (Figures 2D,E).

### Mutation Analysis

The differences in mutated genes between groups with positive or negative MAVS expression were compared, and a landscape map of mutations was constructed (Figure 2C).

### Gene Set Enrichment Analysis and Gene Set Variation Analysis

**FIGURE 3:**
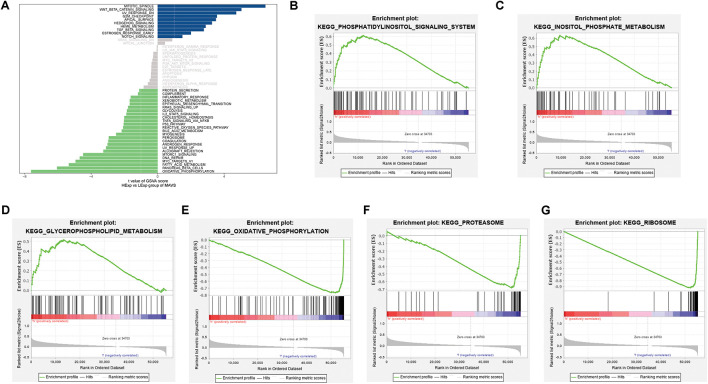
Significant MAVS-related biological pathways in OC obtained by GSEA and GSVA. **(A)** Significant MAVS-related biological pathways in OC obtained by GSVA. **(B–D)** Top three oncological signatures significantly enriched in the MAVS positive expression group identified by GSEA analysis. **(E–G)** Top three oncological signatures significantly enriched in the MAVS negative expression group identified by GSEA analysis.

To explore the potential molecular mechanism and biological functions of MAVS in OC, GSVA, and GSEA were performed for pathway analysis. The results of GSVA revealed that the mitotic spindle, Wnt/β-Catenin signaling, and UV-responsive-Dn were the most significant pathways in the MAVS-positive expression group. Correspondingly, the most involved pathways negatively related to MAVS expression were heme metabolism, apoptosis, and adipogenesis. In addition, the GSEA results showed that phosphatidylinositol signaling, inositol phosphate metabolism, and glycerophospholipid metabolism were statistically significant in the MAVS-positive expression group. Eight KEGG pathways were significantly enriched in the MAVS-negative expression group, namely, oxidative phosphorylation, proteasome, ribosome, and other pathways (Figure 3). These enriched KEGG pathways revealed that MAVS-related molecular changes included phospholipid metabolism and oxidative stress, primarily participating in the membrane state and immune response.

### Construction of the Weighted Gene Co-expression Network Analysis and Functional Enrichment Analysis

**FIGURE 4:**
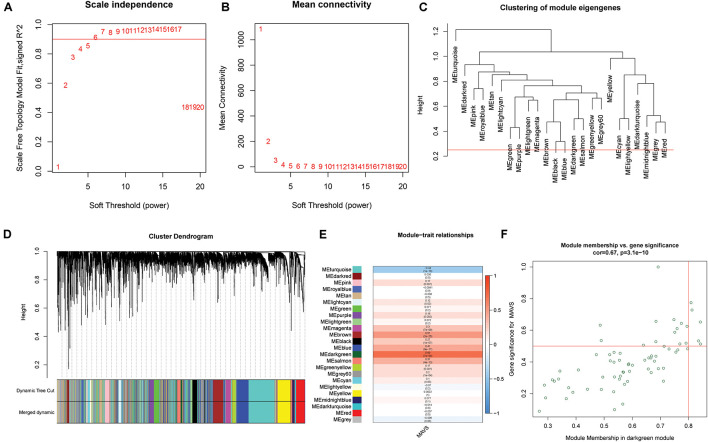
Weighted gene co-expression network (WGCNA) analysis of MAVS-related genes in OC. **(A,B)** Topological network analysis of the optimal soft threshold. **(C)** Similar modules with correlating module eigengenes were merged to form 24 major modules based on a distance threshold cut-off of 0.25. **(D)** Heat map of the correlations between the 24 modules and two different MAVS expression groups. **(E)** Correlation analysis of modules and traits. Dark green module showed maximum correlation with positive MAVS expression traits. **(F)** Intramodular analysis of 69 genes that has the most significant correlations with MAVS expression in the dark green module (correlation = 0.69, *P* = 7e-54).

**FIGURE 5:**
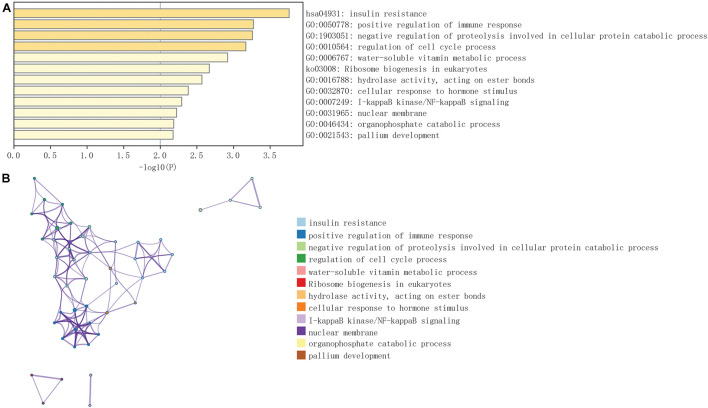
GO and KEGG pathway enrichment analysis of 69 genes in dark green module selected by WGCNA. **(A)** The most significantly enriched pathways of the selected 69 genes (*P* < 0.05). **(B)** Network showed pathways enriched of the selected 69 genes. Different colors of nodes represent different enrichment pathways.

Weighted gene co-expression network analysis was applied to explore the co-expression network of MAVS in ovarian cancer. For such analysis, a suitable soft threshold was selected according to the “sft$power Estimate” function. Twenty-four modules were identified based on TOM matrix detection gene modules. Among all modules, dark green, which represented 69 genes, was the most relevant for MAVS expression (cor = 0.69, *P* = 7e-54), suggesting that genes in the dark green module are highly correlated with MAVS (Figure 4 and Supplementary Table 1). GO and KEGG pathway enrichment analyses were performed to identify the functions of these 69 genes. The results showed that the genes were enriched in the following biological processes: insulin resistance, positive regulation of immune response, and negative regulation of proteolysis involved in cellular protein catabolic process (Figure 5). This finding was consistent with the GSEA and GSVA results, suggesting that MAVS participates in the immune response.

### Mitochondrial Antiviral Signaling Expression Is Correlated With the Immune Infiltration Level in Ovarian Cancer

**FIGURE 6:**
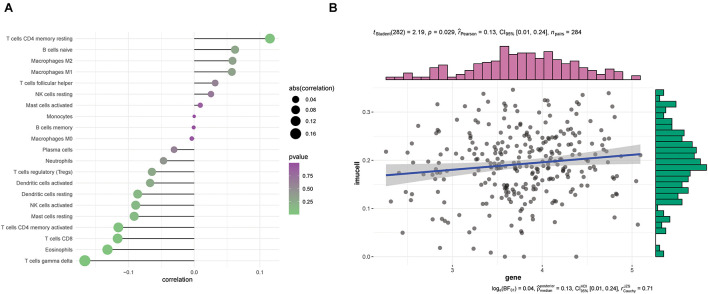
Correlation between MAVS expression and infiltrating immune cells. **(A)** Correlation between MAVS expression and infiltrating immune cells. **(B)** The linear relationship between MAVS expression and the delta gamma T cells is shown.

We performed a synthetic evaluation to explore the role of immune cell infiltration in ovarian cancers using CIBERSORT. The correlation heat map of MAVS expression with immune infiltration levels in ovarian cancers revealed that CD8+ T cells, gamma delta T cells, and eosinophils had a significantly negative correlation with MAVS expression. No association was discovered between MAVS expression and immune cell infiltration (Figure 6A). The linear relationship between MAVS and immune cells is shown in Figure 6B and Supplementary Figure 1.

### Relationships Between Chemosensitivity and Mitochondrial Antiviral Signaling Expression in Ovarian Cancer

**FIGURE 7:**
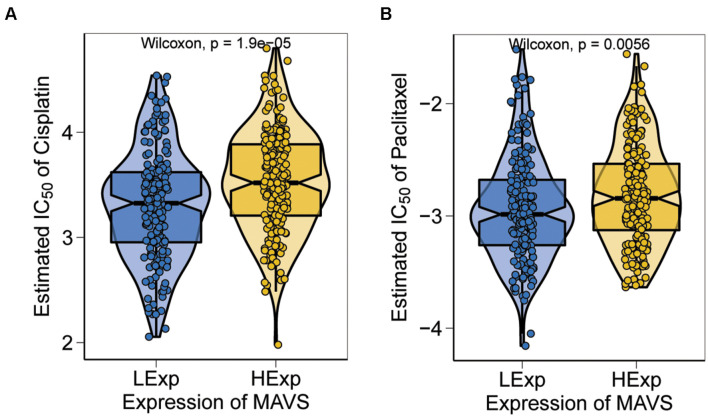
Sensitivity analysis based on MAVS expression. The high MAVS expression group was predicted significantly less respond to cisplatin (*P* = 0.000; **A**) and paclitaxel (*P* = 0.0056; **B**), respectively.

Chemotherapy is one of the most used treatments for ovarian cancer, and chemosensitivity is an important prognostic factor in OC. In the present study, a predictive model of commonly used chemo drugs in ovarian cancer was performed to investigate whether patients with positive MAVS expression are more sensitive to chemotherapy. The results confirmed that the high MAVS expression group was significantly less responsive to cisplatin (*P* = 0.000; Figure 7A) and paclitaxel (*P* = 0.0056; Figure 7B) compared to the low MAVS expression group.

## Discussion

Mitochondrial antiviral signaling is known to be essential for antiviral innate immunity, but it has not been widely studied. In the present study, we identified MAVS as a potential indicator for predicting the prognosis of OC based on 128 ovarian cancer tissues by IHC. We discovered for the first time that high expression of MAVS is closely related to Ki67 expression, pathological stages, and recurrence rate in OC. Moreover, the Kaplan–Meier method indicated a negative correlation between MAVS expression and OC prognosis, namely, overall survival and disease-free survival. Univariate analysis indicated that MAVS expression was negatively associated with overall survival. Together, these results implied that MAVS expression may be a prognostic indicator or a marker for targeted therapy. Based on these observations, we further analyzed underlying functions and mechanisms of MAVS in OC.

First, data from 379 OC samples were downloaded from TCGA database. All samples were divided into high or low expression groups based on the expression of MAVS. A nomogram prediction model further confirmed the prognostic effect of MAVS in OC. According to co-expression analysis, the top five genes positively associated with MAVS were STK35, PANK2, CDS2, ZNF343, and TBC1D20. STK35 belongs to new kinase family 4 (NKF4) in the kinome and regulates the cell cycle transition of endothelial cells. It has been reported that the downregulation of STK35 promotes apoptosis and decreases proliferation by activating caspase 3 and caspase 7 in osteosarcoma cells (Wu et al., 2018). Additionally, STK35 inhibits apoptosis and influences chemoresistance in colorectal cancer (Yang et al., 2020). Moreover, STK35 protein promotes caspase-independent cell death in response to oxidative stress (Yasuda et al., 2012). PANK2 has been reported to be a potential therapeutic target in thyroid cancer (Iacobas et al., 2018). In addition, similar to STK35, PANK2 increases cell death by enhancing oxidative stress in leukemic cells (Liu et al., 2019). In tumor models, CDS2 deficiency suppresses tumor growth by regulating tumor-secreted VEGFA (Zhao et al., 2019). KRAB-containing zinc protein, encoded by ZNF343, is related to cell apoptosis, proliferation, and neoplastic transformation (Urrutia, 2003). TBC1D20 is a negative regulator of Rab1 and is involved in vesicle-mediated transport and metabolism of proteins (Haas et al., 2007). The top five genes negatively related to MAVS were ATP6V0E1, RNF181, OST4, RPL26L1, and TMSB10. It has been reported that some of these genes play a role in tumor proliferation and chemotherapy resistance. However, the potential mechanism has not yet been established, indicating the need for additional research (Thorp et al., 1994; Baroy et al., 2016; Zeng et al., 2020). The results of the co-expression analysis indicated that MAVS participates in tumor progression by regulating genes involved in oxidative stress and metabolic pathways.

To further identify the molecular differences correlated with MAVS expression, we performed GSVA and GSEA to identify potential molecular mechanisms and biological functions. According to the GSVA results, the MAVS-positive expression group was enriched in the mitotic spindle, Wnt/β-Catenin signaling, UV-response-Dn synapse pathway, Hh signaling pathway, and Notch signaling pathway. The Hh pathway is a signaling cascade that is essential for proper development in animals. Recently, accumulating reports have suggested that the Hh signaling pathway promotes tumor invasion, migration and chemotherapy resistance in ovarian cancer (Steg et al., 2012; Zhang H. et al., 2020). The Notch pathway and Wnt pathway are highly conserved and play a biological role in cell proliferation and apoptosis. Various pathway-associated genes are epigenetically regulated in cancers and are related to cancer proliferation, apoptosis and chemoresistance (Vinson et al., 2016; Li et al., 2017; Meurette and Mehlen, 2018; Taciak et al., 2018). Although little is known about the upregulation mechanisms of these pathways in the MAVS-positive expression group, MAVS may influence the prognosis of ovarian cancer by regulating these pathways. The MAVS-negative expression group was significantly enriched in heme metabolism, apoptosis, and adipogenesis. In addition, the GSEA results showed that the phosphatidylinositol signaling system, inositol phosphate metabolism, and glycerophospholipid metabolism were statistically significant in the MAVS-positive expression group. Phospholipids form membrane structures and are recognized in cancers by innate-like T cells, which are essential for initiating the immune response (Zhao et al., 2019). In addition, inositol phosphate metabolism, phosphatidylinositol signaling and glycerophospholipid metabolism are suggested to be essential to immune signaling (Fritsche, 2006; Zhong et al., 2008; Okkenhaug, 2013). Furthermore, oxidative phosphorylation, proteasome, and ribosome pathways were significantly abundant in the MAVS-negative expression group. Inhibition of oxidative phosphorylation (OXPHOS) has been demonstrated to respond to the immune response (Boreel et al., 2021). The proteasome is required for essential immune functions of activated CD4 (+) T cells (Berges et al., 2008). Moreover, the link between ribosome abundance and cell intrinsic immunity has been confirmed (Bianco and Mohr, 2019). These findings revealed that MAVS-related molecular alterations are an immune response. To detect the critical modules most relevant to MAVS expression in OC, WGCNA was performed. GO and KEGG pathway enrichment analyses were performed to identify the functions of the dark green module, comprising 69 genes, which was the most relevant to MAVS expression. The results showed that the biological processes involved in insulin resistance, positive regulation of the immune response, and negative regulation of proteolysis were involved in the cellular protein catabolic process. Initiating insulin resistance is a common process reducing the immune response (Ieronymaki et al., 2019). Similar to the GSEA and GSVA results, these findings suggested that MAVS further participates in the immune response.

Immunoediting is a concept in which the body responds to malignant cells based on the immune system, which has promoted cancer immunotherapy or chemoimmunotherapy in recent years (Coosemans et al., 2015). However, unlike lung cancer immunotherapy, OC immunotherapy is difficult to achieve. A detailed understanding of the cellular immune function of ovarian cancer will contribute to the development of OC immunotherapy. Because MAVS is one of the key innate immune adaptor proteins, we explored the role of MAVS in the immune response in OC by performing a comprehensive analysis using CIBERSORT. The results demonstrated that decreased CD8+ T cells, gamma delta T cells, and eosinophils may be related to MAVS expression in OC. Previous studies have shown that activated eosinophils are essential for guiding T cells into the tumor, leading to tumor eradication (Carretero et al., 2015). Our results supported the possibility that cancers with high MAVS expression have poor prognosis due to a decrease in the immune response regulated by T cells. However, further studies are needed to elucidate the potential mechanisms of these correlations.

Chemoresistance remains a challenge for the treatment of OC, resulting in disease recurrence. Thus, chemosensitivity analysis was performed to investigate the relationship between MAVS expression and commonly used chemotherapeutic drugs in OC. Because platinum-/paclitaxel-based chemotherapy following tumor debulking surgery is still the gold standard treatment for OC, we analyzed whether these drugs are related to MAVS expression. The results indicated that OC with high MAVS expression was significantly less responsive to paclitaxel and cisplatin, suggesting that MAVS inhibitors may enhance the sensitivity of OC to these drugs. Researchers have focused on potential biological mechanisms underlying cisplatin or cisplatin resistance in ovarian cancers, namely, the regulation of chemical transport, DNA repair mechanisms, survival pathways (e.g., MAPK pathway and PI3K/AKT pathway), and tumor suppressors (Brasseur et al., 2017). In the MAVS-positive expression group, the Notch pathway, Hh pathway, and Wnt pathway were upregulated, which have been demonstrated to be related to chemotherapy resistance in ovarian cancer (Khalaf et al., 2021). In addition, MAVS participates in the immune response by itself or via these signaling pathways, which may lead to chemoresistance (Roy and Cowden Dahl, 2018). *In vivo* and *in vitro* experiments are required to validate our results.

## Conclusion

The present study identified MAVS expression as a potential clinical prognostic biomarker or therapeutic target for OC according to immunohistochemistry and prognostic nomogram analyses of OC cases. Bioinformatics analysis of OC within TCGA cohorts demonstrated that MAVS expression potentially contributes to phospholipid-related pathways, which may play a critical role in the immune response by regulating T cells and decreasing the response to paclitaxel and cisplatin. Our study is of great significance for performing MAVS-based experimental validation.

## Data Availability Statement

The datasets presented in this study can be found in online repositories. The names of the repository/repositories and accession number(s) can be found in the article/Supplementary Material.

## Author Contributions

XC designed this project. LC and JH participated in the bioinformatics analysis. BY did IHC analysis. FS and XT drafted the manuscript. All authors listed have made a substantial, direct and intellectual contribution to the work, and approved it for publication.

## Conflict of Interest

The authors declare that the research was conducted in the absence of any commercial or financial relationships that could be construed as a potential conflict of interest.

## Publisher’s Note

All claims expressed in this article are solely those of the authors and do not necessarily represent those of their affiliated organizations, or those of the publisher, the editors and the reviewers. Any product that may be evaluated in this article, or claim that may be made by its manufacturer, is not guaranteed or endorsed by the publisher.

## Footnotes

1
https://portal.gdc.cancer.gov/

2
https://www.cancerrxgene.org/

## Supplementary Material

Supplementary Figure 1 Correlation between MAVS expression and infiltrating immune cells. **(A)** Linear relationship between MAVS expression and CD8 T cells. **(B)** Linear relationship between MAVS expression and eosinophils. **(C)** Linear relationship between MAVS expression and activated memory CD4 T cells. **(D)** Linear relationship between MAVS expression and resting memory CD4 T cells.

Supplementary Table 1 Genes in the dark green module were highly correlated with MAVS by WGCNA analysis.

## References

[B1] Baroy T. Chilamakuri C. S. Lorenz S. Sun J. Bruland ØS. Myklebost O. (2016). Genome analysis of osteosarcoma progression samples identifies FGFR1 overexpression as a potential treatment target and CHM as a candidate tumor suppressor gene. *PLoS One* 11:e0163859. 10.1371/journal.pone.0163859 27685995 PMC5042545

[B2] Berges C. Haberstock H. Fuchs D. Miltz M. Sadeghi M. Opelz G. (2008). Proteasome inhibition suppresses essential immune functions of human CD4+ T cells. *Immunology* 124 234–246. 10.1111/j.1365-2567.2007.02761.x 18217957 PMC2566628

[B3] Bianco C. Mohr I. (2019). Ribosome biogenesis restricts innate immune responses to virus infection and DNA. *Elife* 8:e49551. 10.7554/eLife.49551 PMC6934380 31841110

[B4] Boreel D. F. Span P. N. Heskamp S. Adema G. J. Bussink J. (2021). Targeting oxidative phosphorylation to increase the efficacy of radio- and immune-combination therapy. *Clin. Cancer Res.* 27 2970–2978. 10.1158/1078-0432.ccr-20-3913 33419779

[B5] Brasseur K. Gevry N. Asselin E. (2017). Chemoresistance and targeted therapies in ovarian and endometrial cancers. *Oncotarget* 8 4008–4042. 10.18632/oncotarget.14021 28008141 PMC5354810

[B6] Carretero R. Sektioglu I. M. Garbi N. Salgado O. C. Beckhove P. Hämmerling G. J. (2015). Eosinophils orchestrate cancer rejection by normalizing tumor vessels and enhancing infiltration of CD8(+) T cells. *Nat. Immunol.* 16 609–617. 10.1038/ni.3159 25915731

[B7] Chen L. Hou J. Zeng X. Guo Q. Deng M. Kloeber J. A. (2021). LRRK2 inhibition potentiates PARP inhibitor cytotoxicity through inhibiting homologous recombination-mediated DNA double strand break repair. *Clin. Transl. Med.* 11:e341. 10.1002/ctm2.341 PMC7908045 33784003

[B8] Coosemans A. Baert T. Vergote I. (2015). A view on dendritic cell immunotherapy in ovarian cancer: how far have we come? *Facts Views Vis. Obgyn.* 7 73–78. 25897374 PMC4402447

[B9] Fritsche K. (2006). Fatty acids as modulators of the immune response. *Annu. Rev. Nutr.* 26 45–73. 10.1146/annurev.nutr.25.050304.092610 16848700

[B10] Haas A. K. Yoshimura S. Stephens D. J. Preisinger C. Fuchs E. Barr F. A. (2007). Analysis of GTPase-activating proteins: Rab1 and Rab43 are key Rabs required to maintain a functional Golgi complex in human cells. *J. Cell Sci.* 120(Pt 17) 2997–3010. 10.1242/jcs.014225 17684057

[B11] Iacobas D. A. Tuli N. Y. Iacobas S. Rasamny J. K. Moscatello A. Geliebter J. (2018). Gene master regulators of papillary and anaplastic thyroid cancers. *Oncotarget* 9 2410–2424. 10.18632/oncotarget.23417 29416781 PMC5788649

[B12] Ieronymaki E. Daskalaki M. G. Lyroni K. Tsatsanis C. (2019). Insulin signaling and insulin resistance facilitate trained immunity in macrophages through metabolic and epigenetic changes. *Front. Immunol.* 10:1330. 10.3389/fimmu.2019.01330 31244863 PMC6581697

[B13] Kaneda Y. (2013). The RIG-I/MAVS signaling pathway in cancer cell-selective apoptosis. *Oncoimmunology* 2:e23566. 10.4161/onci.23566 PMC3654583 23734313

[B14] Khalaf K. Hana D. Chou J. T. Singh C. Mackiewicz A. Kaczmarek M. (2021). Aspects of the tumor microenvironment involved in immune resistance and drug resistance. *Front. Immunol.* 12:656364. 10.3389/fimmu.2021.656364 34122412 PMC8190405

[B15] Li L. Tang P. Li S. Qin X. Yang H. Wu C. (2017). Notch signaling pathway networks in cancer metastasis: a new target for cancer therapy. *Med. Oncol.* 34:180. 10.1007/s12032-017-1039-6 28918490

[B16] Liu Y. Cheng Z. Li Q. Pang Y. Cui L. Qian T. (2019). Prognostic significance of the PANK family expression in acute myeloid leukemia. *Ann. Transl. Med.* 7:261. 10.21037/atm.2019.05.28 31355228 PMC6614324

[B17] Matsushima-Miyagi T. Hatano K. Nomura M. Li-Wen L. Nishikawa T. Saga K. (2012). TRAIL and Noxa are selectively upregulated in prostate cancer cells downstream of the RIG-I/MAVS signaling pathway by nonreplicating Sendai virus particles. *Clin. Cancer Res.* 18 6271–6283. 10.1158/1078-0432.ccr-12-1595 23014529

[B18] Meurette O. Mehlen P. (2018). Notch signaling in the tumor microenvironment. *Cancer Cell* 34 536–548. 10.1016/j.ccell.2018.07.009 30146333

[B19] Okkenhaug K. (2013). Signaling by the phosphoinositide 3-kinase family in immune cells. *Annu. Rev. Immunol.* 31 675–704. 23330955 10.1146/annurev-immunol-032712-095946 PMC4516760

[B20] Pythoud C. Rothenberger S. Martínez-Sobrido L. de la Torre J. C. Kunz S. (2015). Lymphocytic choriomeningitis virus differentially affects the virus-induced type I interferon response and mitochondrial apoptosis mediated by RIG-I/MAVS. *J. Virol.* 89 6240–6250. 10.1128/jvi.00610-15 25833049 PMC4474305

[B21] Roy L. Cowden Dahl K. D. (2018). Can stemness and chemoresistance be therapeutically targeted via signaling pathways in ovarian cancer? *Cancers (Basel)* 10:241. 10.3390/cancers10080241 30042330 PMC6116003

[B22] Siegel R. L. Miller K. D. Fuchs H. E. Jemal A. (2021). Cancer statistics, 2021. *CA Cancer J. Clin.* 71 7–33. 33433946 10.3322/caac.21654

[B23] Steg A. D. Katre A. A. Bevis K. S. Ziebarth A. Dobbin Z. C. Shah M. M. (2012). Smoothened antagonists reverse taxane resistance in ovarian cancer. *Mol. Cancer Ther.* 11 1587–1597. 10.1158/1535-7163.mct-11-1058 22553355 PMC3392529

[B24] Taciak B. Pruszynska I. Kiraga L. Bialasek M. Krol M. (2018). Wnt signaling pathway in development and cancer. *J. Physiol. Pharmacol.* 69 185–196. 10.26402/jpp.2018.2.07 29980141

[B25] Thorp K. Scott P. R. Henshaw C. J. Watt N. J. (1994). Extensive anterior subcutaneous oedema associated with thymic lymphosarcoma in a 30-month-old heifer. *Vet. Rec.* 135 530–531. 10.1136/vr.135.22.530 7605433

[B26] Urrutia R. (2003). KRAB-containing zinc-finger repressor proteins. *Genome Biol.* 4:231. 10.1186/gb-2003-4-10-231 PMC328446 14519192

[B27] Vinson K. E. George D. C. Fender A. W. Bertrand F. E. Sigounas G. (2016). The Notch pathway in colorectal cancer. *Int. J. Cancer* 138 1835–1842. 10.1002/ijc.29800 26264352

[B28] Wu Z. Liu J. Hu S. Zhu Y. Li S. (2018). Serine/threonine kinase 35, a target gene of STAT3, regulates the proliferation and apoptosis of osteosarcoma cells. *Cell Physiol. Biochem.* 45 808–818. 10.1159/000487172 29414823

[B29] Yang H. Zhu J. Wang G. Liu H. Zhou Y. Qian J. (2020). STK35 is ubiquitinated by NEDD4L and promotes glycolysis and inhibits apoptosis through regulating the AKT signaling pathway, influencing chemoresistance of colorectal cancer. *Front. Cell. Dev. Biol.* 8:582695. 10.3389/fcell.2020.582695 33117809 PMC7578231

[B30] Yasuda Y. Miyamoto Y. Yamashiro T. Asally M. Masui A. Wong C. (2012). Nuclear retention of importin alpha coordinates cell fate through changes in gene expression. *EMBO J.* 31 83–94. 10.1038/emboj.2011.360 21964068 PMC3252573

[B31] Zeng J. Yang X. Yang L. Li W. Zheng Y. (2020). Thymosin beta10 promotes tumor-associated macrophages M2 conversion and proliferation via the PI3K/Akt pathway in lung adenocarcinoma. *Respir. Res.* 21:328. 10.1186/s12931-020-01587-7 PMC7754581 33349268

[B32] Zhang H. Hu L. Cheng M. Wang Q. Hu X. Chen Q. (2020). The Hedgehog signaling pathway promotes chemotherapy resistance via multidrug resistance protein 1 in ovarian cancer. *Oncol. Rep.* 44 2610–2620. 10.3892/or.2020.7798 33125122 PMC7640363

[B33] Zhang W. Gong J. Yang H. Wan L. Peng Y. Wang X. (2020). The mitochondrial protein MAVS stabilizes p53 to suppress tumorigenesis. *Cell Rep.* 30 725–738 e4. 31968249 10.1016/j.celrep.2019.12.051

[B34] Zhao W. Cao L. Ying H. Zhang W. Li D. Zhu X. (2019). Endothelial CDS2 deficiency causes VEGFA-mediated vascular regression and tumor inhibition. *Cell Res.* 29 895–910. 10.1038/s41422-019-0229-5 31501519 PMC6889172

[B35] Zhong X. P. Guo R. Zhou H. Liu C. Wan C. K. (2008). Diacylglycerol kinases in immune cell function and self-tolerance. *Immunol. Rev.* 224 249–264. 10.1111/j.1600-065x.2008.00647.x 18759932 PMC3342643

