# Supplementary figures and images for: An Analysis Regarding the Prognostic Significance of MAVS and Its Underlying Biological Mechanism in Ovarian Cancer
Source: Front Cell Dev Biol. 2021 Oct 14;9:728061. doi: 10.3389/fcell.2021.728061 (PMC8551630; doi:10.3389/fcell.2021.728061)

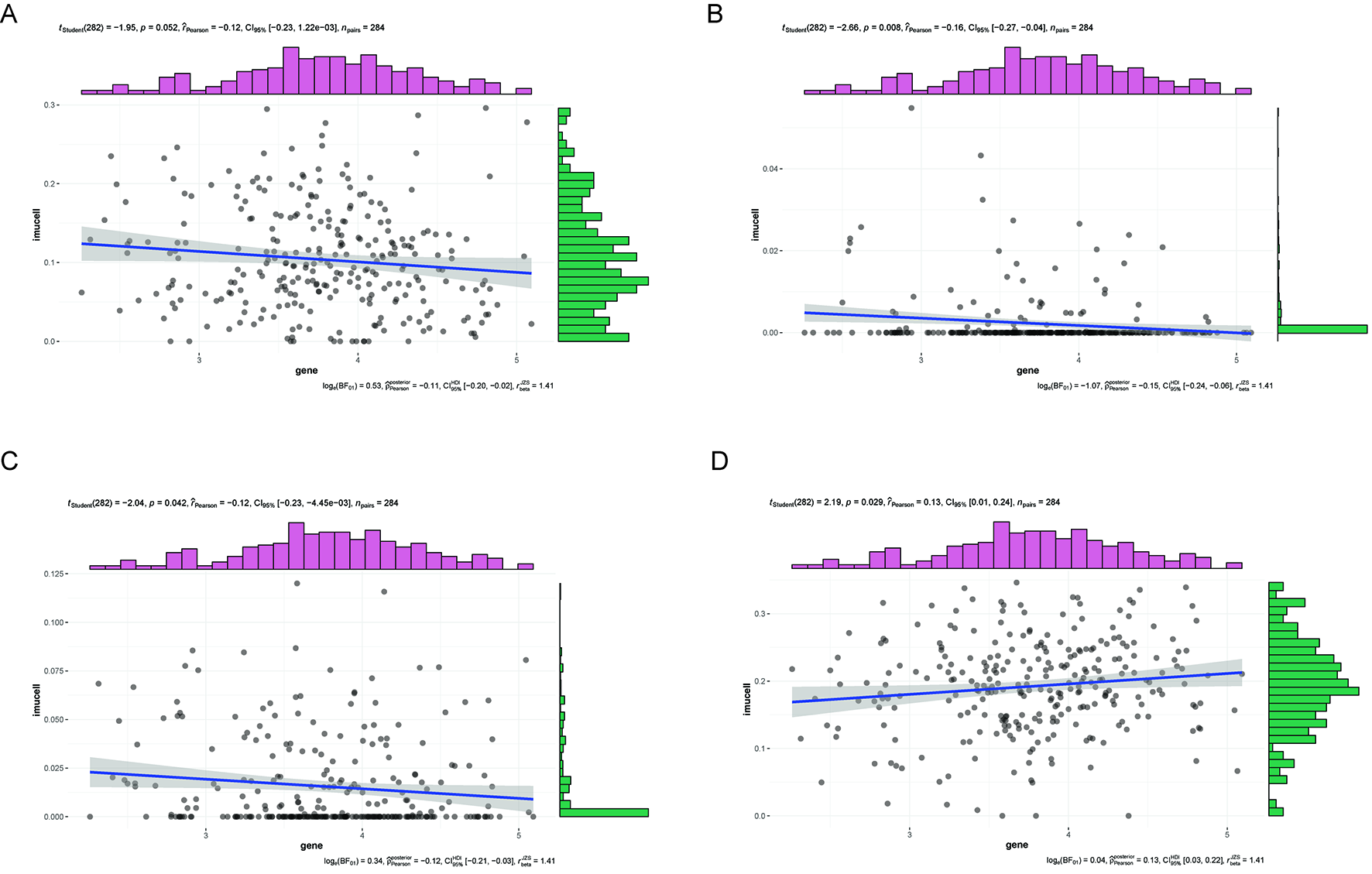

Supplement: Supplementary Figure 1 — Correlation between MAVS expression and infiltrating immune cells. (A) Linear relationship between MAVS expression and CD8 T cells. (B) Linear relationship between MAVS expression and eosinophils. (C) Linear relationship between MAVS expression and activated memory CD4 T cells. (D) Linear relationship between MAVS expression and resting memory CD4 T cells. [file Image_1.tif]
